# Raman Spectroscopy of Liquid-Based Cervical Smear Samples as a Triage to Stratify Women Who Are HPV-Positive on Screening

**DOI:** 10.3390/cancers13092008

**Published:** 2021-04-22

**Authors:** Damien Traynor, Cara M. Martin, Christine White, Stephen Reynolds, Tom D’Arcy, John J. O’Leary, Fiona M. Lyng

**Affiliations:** 1 Centre for Radiation and Environmental Science, FOCAS Research Institute, Technological University Dublin, Kevin St, D08 NF82 Dublin, Ireland; damien.traynor@tudublin.ie; 2 School of Physics & Clinical & Optometric Sciences, City Campus, Technological University Dublin, Kevin Street, D08 NF82 Dublin, Ireland; 3 Discipline of Histopathology, University of Dublin Trinity College, D08 NHY1 Dublin, Ireland; cara.martin@tcd.ie (C.M.M.); chwhite@tcd.ie (C.W.); stephen.reynolds@tcd.ie (S.R.); olearyjj@tcd.ie (J.J.O.); 4 CERVIVA Molecular Pathology Research Laboratory, The Coombe Women and Infants University Hospital, D08 XW7X Dublin, Ireland; 5 The Trinity St. James’s Cancer Institute, D08 NHY1 Dublin, Ireland; 6 Department of Obstetrics and Gynaecology, Coombe Women and Infants University Hospital, D08 XW7X Dublin, Ireland; tomdarcy@coombe.ie

**Keywords:** Raman spectroscopy, cytology, HPV, biomarkers, ThinPrep, exfoliated cells, cervical cancer, cervical precancer

## Abstract

**Simple Summary:**

Persistent high-risk human papillomavirus (HPV) infection can lead to cervical precancer and cancer. Recently, HPV testing has been introduced for primary cervical screening, but the HPV DNA test cannot distinguish between transient and persistent HPV infection. Thus, there is an unmet clinical need to develop a new test to identify women with a high-risk persistent HPV infection. Raman spectra were recorded from cervical smear samples (*n* = 60) and, on the basis of HPV DNA and HPV mRNA test results, a classifier was developed to identify persistent HPV infection. A further blinded independent test set (*n* = 14) was used to validate the model, and sensitivity of 90% and specificity of 100% were achieved. Improved triage would allow women with a high-risk persistent HPV infection to be referred for immediate treatment, while women with a low-risk transient infection could avoid overtreatment.

**Abstract:**

The role of persistent high-risk human papillomavirus (HPV) infection in the development of cervical precancer and cancer is now well accepted, and HPV testing has recently been introduced for primary cervical screening. However, the low specificity of HPV DNA testing can result in large numbers of women with an HPV-positive result, and additional triage approaches are needed to avoid over-referral to colposcopy and overtreatment. The aim of this study was to assess Raman spectroscopy as a potential triage test to discriminate between transient and persistent HPV infection. HPV DNA status and mRNA status were confirmed in ThinPrep^®^ cervical samples (*n* = 60) using the Cobas 4800 and APTIMA HPV test, respectively. Raman spectra were recorded from single-cell nuclei and subjected to partial least squares discriminant analysis (PLSDA). In addition, the PLSDA classification model was validated using a blinded independent test set (*n* = 14). Sensitivity of 85% and specificity of 92% were achieved for the classification of transient and persistent HPV infection, and this increased to 90% sensitivity and 100% specificity when mean sample spectra were used instead of individual cellular spectra. This study showed that Raman spectroscopy has potential as a triage test for HPV-positive women to identify persistent HPV infection.

## 1. Introduction

Cervical cancer ranks fourth for both incidence and mortality with an estimated 570,000 cases and 311,000 deaths worldwide in 2018 [1]. In order to achieve the World Health Organization’s call to action to eliminate cervical cancer, improved screening and early detection are required [2,3].

Persistent infection with high-risk human papillomavirus (HPV) is accepted as the major cause of the development of cervical precancer and cancer [4]. Over 100 different types of HPV have been identified, and 14 are considered as high-risk HPV types (hrHPV 16, 18, 31, 33, 35, 39, 45, 51, 52, 56, 58, 59, 66, and 68) [5]. HPV is a common sexually transmitted infection, and most HPV infections are transient and are resolved in 1–2 years [6]. Persistent hrHPV infection, however, followed by integration of the HPV genome into the host chromosomes, results in production of E6/E7 messenger RNA (mRNA) transcripts and E6/E7 oncoproteins causing cell-cycle deregulation, which can lead to precancer (cervical intraepithelial neoplasia (CIN)) [7]. This persistent form of HPV infection can be termed a transcriptionally active HPV infection.

HPV testing has recently replaced cytology as the primary cervical screening method in many countries, including Ireland [8]. HPV testing has a higher sensitivity than cytology for detection of high-grade cervical precancer (CIN2/3). However, HPV DNA testing is limited by its lower specificity to cytology; thus, additional approaches are required to triage HPV DNA-positive women to avoid over-detection of clinically insignificant, transient HPV infections as these can result in over-referral to colposcopy and overtreatment [9].

Cytology-based triage, with and without HPV16/18 genotyping, is generally used to identify women with low-grade squamous intraepithelial lesions (LSIL) and high-grade squamous intraepithelial lesions (HSIL). However, cytology alone has a sensitivity of only 59–70% for detection of HSIL due to sampling, technical, and/or inter-observer errors mainly associated with the subjectivity of the morphological assessment [10].

Thus, there is an unmet need for additional triage tests that can identify disease-specific biomarkers. A number of molecular biomarkers have recently been investigated to detect transforming HPV infections including E6/E7 mRNA, methylation markers, miRNAs, and protein biomarkers, such as p16INK4a, Ki67, minichromosome maintenance protein 2 (MCM), and topoisomerases type IIa (TOP2A) [11].

As an alternative approach to single molecular biomarkers, Raman spectroscopy can provide a biochemical fingerprint of a cell or tissue [12,13] and, thus, offers an opportunity to capture molecular changes in one test. Many studies have shown the potential of Raman spectroscopy for noninvasive, objective detection of cervical cancer and precancer in cells and tissues [14,15,16,17,18,19,20,21,22,23,24,25,26,27,28,29,30], but there are limited studies on detection of HPV infection.

Jess et al. [31] showed that Raman microspectroscopy could distinguish between primary human keratinocytes (PHK) and PHK cells expressing the E7 gene of HPV16 and between PHK cells and cervical cancer cells expressing HPV16 (CaSki). Both infrared absorption and Raman spectroscopy were shown to distinguish between am HPV-negative cervical cancer cell line (C33a) and cervical cancer cell lines with different HPV copy number (SiHa, HeLa, and CaSki) as a function of the spectral profile of the cells [32]. Vargis et al. [16] showed discrimination between different HPV-negative and HPV-positive cell lines and cytology samples using Raman spectroscopy. More recently, biochemical changes due to HPV infection in HSIL cells were shown to be more marked than biochemical changes due to the menstrual cycle or the use of hormonal contraceptives [29].

To our knowledge, no studies to date have investigated the potential of Raman spectroscopy for detection of transcriptionally active HPV infection. Thus, the main aim of this study was to assess Raman spectroscopy as a potential triage test for HPV-positive cervical samples. Raman spectra were recorded from liquid-based cytology (LBC) samples prepared as ThinPrep slides (*n* = 60); then, on the basis of HPV DNA and HPV mRNA test results, a classification model was developed to identify transcriptionally active HPV infections. A further blinded independent test set (*n* = 14), which was completely separate from the training set, was utilized to validate the model, and the results were compared to cytology, histopathology, and HPV DNA and mRNA test results.

## 2. Materials and Methods

### 2.1. Sample Collection

Residual material from cervical smear samples collected in PreservCyt solution was obtained from women attending the colposcopy clinic at the Coombe Women and Infants University Hospital (CWIUH), Dublin, Ireland, for a routine colposcopy visit. Women gave written informed consent for their sample to be used for this research study. Ethical approval was obtained from the CWIUH Research Ethics Committee (Study No. 28-2014). A subset of samples was obtained from the residual cervical smear samples from women enrolled in the CERVIVA HPV Primary Screening Trial. Ethical approval for this was obtained from the ICGP Research Ethics Committee. This study is part of a larger study underway within CERVIVA and, thus, samples were specifically selected that were HPV DNA/mRNA-positive or HPV DNA-positive only. A total of 60 cervical LBC samples were used for this study to develop the classification model (Table 1). HPV mRNA-positive samples represented the transcriptionally active form of infection (*n* = 30) and HPV DNA-positive mRNA-negative samples represented the non-transcriptionally active form (*n* = 30).

HPV DNA status was confirmed using the HPV Cobas 4800 test which detects the presence of hrHPV DNA. This test detects HPV16 and HPV18 separately, as well as a pool of 12 other high-risk types (HPV 31, 33, 35, 39, 45, 51, 52, 56, 58, 59, 66, and 68).

HPV mRNA status was confirmed using the Aptima HPV Assay. The Aptima HPV assay is a target amplification nucleic acid probe test for the in vitro qualitative detection of E6/E7 viral messenger RNA (mRNA) from 14 high-risk types of HPV (16/18/31/33/35/39/45/51/52/56/58/59/66/68). The Aptima HPV assay does not discriminate between the 14 high-risk types.

A further set of blinded samples (*n* = 14), selected as above but completely separate from the training set, was used as a test set to validate the classification model and consisted of a mixture of non-transcriptionally active and transcriptionally active HPV infected samples.

### 2.2. ThinPrep Slide Preparation

Samples were prepared for Raman spectroscopy using the ThinPrep 2000 processor (Hologic Inc., Marlborough, MA, USA). The ThinPrep processor homogenizes the sample by spinning the filter, creating shear forces that break up any clumped material (blood, mucin and non-diagnostic material). The cells are then transferred onto the TransCyt filter and transferred onto a glass slide to produce a circular monolayer of cells approximately 20 mm in diameter. The slide is then ejected into a fixative bath of 95% ethanol.

The slides then underwent a pretreatment step to remove any molecular contamination by hemoglobin, which obscures several features of the cellular spectrum as described previously [28]. Briefly, slides were treated with a 30% solution of H_2_O_2_ at room temperature for 3 min, followed by a 70% solution of industrial methylated spirits (IMS) for 3 min, followed by multiple dips into 100% IMS to remove any remaining cellular debris and H_2_O_2_, before being air-dried.

### 2.3. Raman Spectroscopy

**Figure 1:**
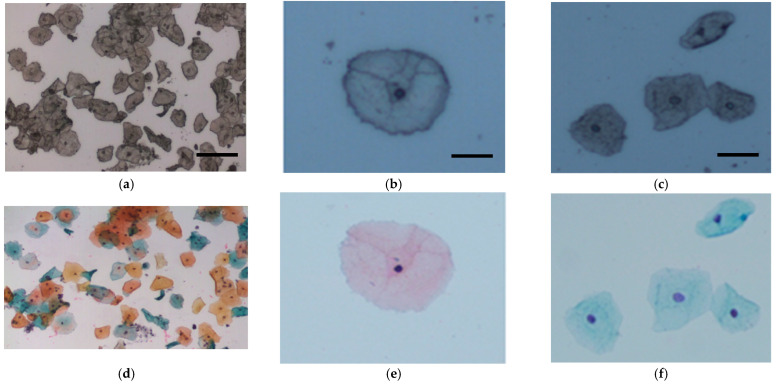
Representative cervical cells from (**a**) unstained and (**d**) stained Pap smears (bar = 50 μm). Superficial cells (**b**) before and (**e**) after staining (bar = 20 μm) and intermediate cells (**c**) before and (**f**) after staining (bar = 20 μm).

A HORIBA Jobin Yvon XploRA system (Villeneuve d’Ascq, France), with a 532 nm diode laser source, which incorporates an Olympus microscope BX41 equipped with a X100 objective (MPlan, Olympus, NA = 0.9) was utilized to record Raman spectra. Laser power was set to 100% resulting in 16 mW at the objective, and the confocal hole, coupled to a slit aperture of 100 μm, was set at 100 μm. Raman signals were detected using a spectrograph with a 1200 g/mm grating coupled to a charge-coupled device (Andor, 1024 × 256 pixels) and the spectrometer was controlled by Labspec V6.0 software. The spectrometer was calibrated daily using the 520 cm^−1^ line of silicon before sample acquisition. Calibration spectra of 1,4-bis(2-methylstyryl) benzene (Sigma Aldrich, Arklow, Ireland) were also recorded at the time of each sample acquisition. All spectra were subsequently wavenumber calibrated using in-house developed procedures in Matlab v.9.3 (Mathworks Inc., Natick, MA, USA). For sample acquisition, a Raman spectrum was acquired for each cell from the nucleus in the fingerprint region, 400 to 1800 cm^−1^, with an integration time of 30 s averaged over two accumulations. As spectra from the cell nucleus have been found to be more reproducible and consistent than spectra from the cell cytoplasm [25], only nuclear spectra were recorded. Where possible, spectra were recorded from at least 20–30 randomly selected morphologically normal superficial and intermediate cells from each unstained Pap smear, as shown in Figure 1. This resulted in a total of 1500 spectra for the training set of samples (750 spectra for samples 1–30 and 750 spectra for samples 31–60) and a total of 350 spectra for the blinded test set. The time taken to record spectra from 20–30 cells per sample was approximately 30 min.

### 2.4. Data Preprocessing and Analysis

Data preprocessing was performed using Matlab software (Mathworks Inc., Natick, MA, USA) and in-house scripts, including smoothing (Savitzky-Golay K = 5, K = 13), baseline correction (rubberband), and vector normalization. A non-negative least-squares (NNLS) method was used for glass correction as described previously [27,29]. The data were mean-centered and partial least squares discriminant analysis (PLS-DA) using the PLS toolbox (Eigenvector Research, Washington, DC, USA) in the Matlab (Mathworks Inc., Natick, MA, USA) environment was used to build classification models. Leave-one-patient-out cross-validation (LOPOCV) was used, which involved removing all data from one patient sample from the model and repeating this process until all patient samples were left out once.

For further validation of the model, a blinded independent test set was employed (*n* = 14). Firstly, all spectra were tested individually and, secondly, the mean spectrum of each patient sample was tested. The positive class comprised samples with a transcriptionally active HPV infection because the aim of screening is to identify patients most at risk who will require further treatment. The negative class comprised samples with a non-transcriptionally active HPV infection because they are deemed to be at a lower risk of developing cervical cancer in the next 5 years and can return to routine screening.

## 3. Results

**Figure 2:**
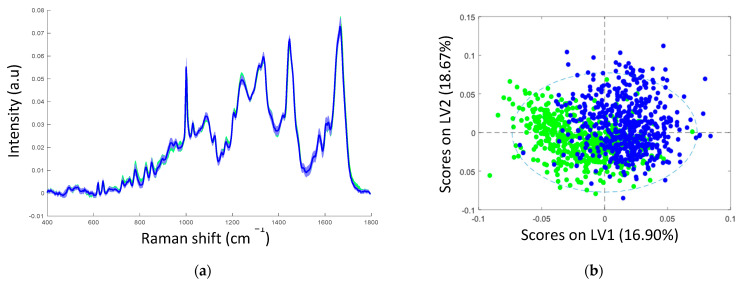

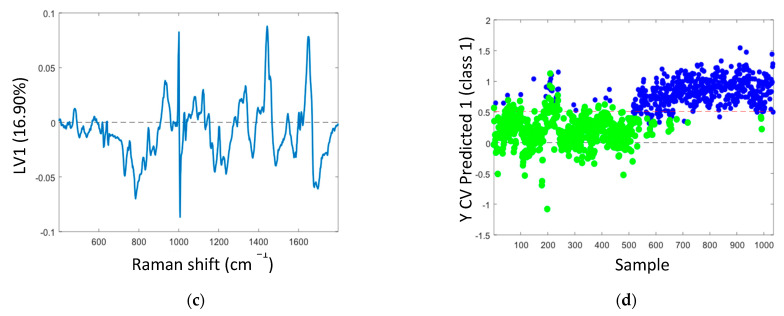
(**a**) Mean spectra of samples with non-transcriptionally active HPV infection (green, *n* = 750 spectra) and transcriptionally active HPV infection (blue, *n* = 750 spectra); shading indicates the standard deviation; (**b**) latent variable (LV) score scatter plot of LV1 and LV2 of samples with non-transcriptionally active HPV infection (green) and transcriptionally active HPV infection (blue); (**c**) LV1 loadings; (**d**) PLSDA plot.

The mean Raman spectra of samples with non-transcriptionally active and transcriptionally active HPV infection showed similar spectral profiles, as can be seen in Figure 2a. Table 2 illustrates the tentative Raman band assignments used in the present study. Differences were noted at Raman peak positions 782 cm^−1^ (nucleic acids), 1238 cm^−1^ (amide III), and 1670 cm^−1^ (amide I). A classification model based on the Raman spectral data was developed using PLS-DA with LOPOCV. The scatter plot shows that latent variable LV1 mainly contributed to the differentiation of samples with non-transcriptionally active and transcriptionally active HPV infection (Figure 2b). The loadings from LV1 are shown in Figure 2c and show that discrimination is based around Raman peaks at 482 (glycogen), 727 (nucleic acids), 782 (nucleic acids), 826 (nucleic acids), 852 (glycogen), 937 (glycogen), 1082 (glycogen), 1123 (glycogen), 1152 (proteins), 1334 (glycogen), 1380 (glycogen), 1238 (amide III), 1450 (proteins/lipids), 1485 (nucleic acids), 1580 (nucleic acids), 1642 (proteins), and 1670 cm^−1^ (amide I). Although a shift around 1002 cm^−1^ (phenylalanine) can indicate calibration issues, a rigorous wavenumber calibration procedure was carried out; therefore, it is likely that this is a protein-related difference between the two groups. A sensitivity of 88% and a specificity of 88% were achieved by PLSDA classification with LOPOCV (Figure 2d), indicating that Raman spectroscopy can distinguish non-transcriptionally active HPV infection from transcriptionally active HPV infection.

The next step was to validate the results with a blinded test set. The results are shown in Table 3 and show the result of (1) testing all sample spectra individually and (2) testing the mean spectrum of each patient sample. Nine out of 10 transcriptionally active samples and four out of four non-transcriptionally active samples were classified correctly using both types of testing regime. A sensitivity of 85% and a specificity of 92% were achieved for testing all sample spectra individually, and this increased to 90% and 100% for sensitivity and specificity, respectively, when the mean spectrum of each sample was tested. In both cases, sample 010 was incorrectly classified. When each cellular spectrum was tested individually, 10 out of 16 of the individual cellular spectra were classified incorrectly as non-transcriptionally active when they were from a transcriptionally active sample. When the mean spectrum of sample 010 as a whole was tested, it was again classified incorrectly as a non-transcriptionally active sample.

The Raman classification results, together with the cytology, histology, HPV DNA, and HPV mRNA results, are shown in Table 4 for the blinded test set samples. Samples 001–009, which were classified correctly by Raman spectroscopy as transcriptionally active samples, were HSIL on cytology and, apart from sample 005, were confirmed as CIN2/3 on histology. In addition, each of these samples (001–009) was both HPV DNA-positive and mRNA-positive. Similarly, samples 011–014, which were classified correctly as non-transcriptionally active samples, were either negative or LSIL on cytology and negative on histology. Although these samples 011–014 were HPV DNA-positive, each one was HPV mRNA-negative. Sample 010, which was classified incorrectly by Raman spectroscopy as a non-transcriptionally active sample, was LSIL on cytology, CIN1 on histology, and both HPV DNA-positive and mRNA-positive.

## 4. Discussion

In this study, the aim was to investigate the use of Raman spectroscopy as a triage test for HPV-positive cases. Initially, Raman spectra were recorded from a training set consisting of cervical LBC samples (*n* = 60). Samples were specifically selected for the training set as HPV-positive and high-grade HSIL cytology, and half of the samples (*n* = 30) tested positive for HPV mRNA. The mean Raman spectra and LV1 loadings showed that the discrimination was mostly based around increased nucleic acids (727, 781, 826, 1485, and 1580 cm^−1^), decreased glycogen (482, 852, 937, 1082, 1123, 1334, and 1380 cm^−1^), and changes in protein features (1152, 1240, 1450, 1640, and 1670 cm^−1^), indicating increased proliferation and altered protein expression due to the overexpression of E6/E7 viral proteins in the samples with a transcriptionally active HPV infection. These discriminating spectral features are consistent with previous studies on exfoliated cells showing discrimination between samples with negative cytology and HSIL cytology [25,27,28,30,34] and between samples with negative cytology and HPV DNA-positive HSIL samples [29]. PLSDA classification with LOPOCV achieved a sensitivity of 88% and a specificity of 88% in distinguishing non-transcriptionally active HPV infection from transcriptionally active HPV infection.

This classification model was further tested using a blinded test set (*n* = 14). Raman spectra were recorded from this test set, and both individual cellular spectra and mean sample spectra were used to test the model. Both methods provided successful validation of the classification model with nine out of 10 samples with a transcriptionally active HPV infection and four out of four samples with a non-transcriptionally active HPV infection classified correctly. Sensitivity of 85% and specificity of 92% were achieved using individual cellular spectra, and this increased to 90% sensitivity and 100% specificity using mean sample spectra. The variability within the dataset was reduced by utilizing the mean spectra, resulting in an increase in sensitivity and specificity.

The need to record multiple cellular spectra from the same sample is highlighted in the data for sample 002, as, although this sample was classified correctly using individual cellular spectra, there was only one cellular spectrum in the difference. This suggests that not every cell within an HPV-infected sample will exhibit the associated biochemical signature of a persistent HPV infection. Factors such as sampling technique, the size of the sampled area, or the presence of HPV mRNA in every cell tested could account for this. Mean spectral data can reduce the variability in each sample, and it is expected that correct classification should be achieved if the mean sample spectrum shows the biochemical changes associated with a transcriptionally active HPV infection.

All the transcriptionally active samples tested in this study were reported as HSIL; thus, currently, it is not possible to determine if the cytology grade (LSIL or HSIL) affects the number of cells classified into each category. LSIL samples that are HPV DNA/mRNA-positive need to be included in future studies to see if this affects the correct classification of individual cellular spectra.

Notably, very good agreement was observed among the cytology, histology, HPV tests, and the Raman classification result. The majority of samples that were classified as having a transcriptionally active HPV infection were both HPV DNA-positive and mRNA-positive and were reported as HSIL on cytology. The cytology result was confirmed as CIN2/3 by histology in all but one sample. Four out of five samples that were classified as having a non-transcriptionally active HPV infection were HPV DNA-positive but mRNA-negative and were reported as either negative or LSIL on cytology. All four samples were negative on histology. Interestingly, the sample that was classified incorrectly as having a non-transcriptionally active HPV infection was HPV DNA-positive and mRNA-positive but was reported as LSIL on cytology and confirmed as CIN1 on histology. A limitation of our study is that the training set consisted only of HSIL cases; hence, future work would need to include LSIL cases to fully train the classification model.

An additional limitation of this study is the relatively low number of samples with a non-transcriptionally active HPV infection in the blinded test set. As the main source of sample recruitment was a colposcopy clinic, where most patients were referred for further assessment of a HSIL cytology result, the majority of patients had a transcriptionally active HPV infection. This proof-of-principle study provides sufficient evidence to warrant a larger study in a screening population where HPV is the primary screening test and the clinical value of Raman spectroscopy for HPV triage can be realized. This would provide a larger sample base of samples with non-transcriptionally active HPV infection for testing compared to colposcopy-based recruitment.

## 5. Conclusions

In conclusion, this study showed that Raman spectroscopy has potential as a triage test for HPV-positive women to identify transcriptionally active HPV infections, although further work is necessary in a HPV primary screening setting. Unnecessary referral of women with a non-transcriptionally active HPV infection for cytological evaluation could be prevented by an improved triage test, while women with an increased risk of disease are referred to colposcopy. Women most at risk can be identified using a Raman spectroscopy-based test without the subjectivity of morphological assessment. The method can be performed on the same initial Pap test, and, if required, cytology and/or immunocytochemistry for molecular biomarkers can be carried out following the Raman spectral analysis.

## Figures and Tables

**Table 1:** Summary of training set of samples, including cytology, HPV DNA, and HPV mRNA test results.

Scheme	Cytology	HPV DNA	HPV mRNA	Transcriptionally Active HPV Infection
1–30	HSIL	positive	negative	no
31–60	HSIL	positive	positive	yes

HSIL: high-grade squamous intraepithelial lesion.

**Table 2:** Tentative peak assignments [33].

Raman Peak Position (cm^−1^)	Proteins	Lipids	Carbohydrates	Nucleic Acids
482			Glycogen	
577			Glycogen	
622	C–C twist Phe			
643	C–C twist Tyr			
727	CH_2_ def	C–C head		A
752	Sym br. Trp			
781				U, C, T ring br
826	Out of Plane ring br. Tyr			PO2 a.str
851	Ring br. Tyr, C–C str. Pro			
852			Glycogen	
937			Glycogen	
985		C–C head		
1002	Sym. Ring br. Phe			
1033	C–H in plane Phe, C–C str			
1060	C–N str			
1082	C–N str	Chain C–C str	C–O str, Glycogen	
1096		Chain C–C str	C–C str	
1123	C–N str	Chain C–C str	C–O str, Glycogen	
1152	C–N str			
1207	C–C_6_H_5_ str. Phe, Trp			
1238	C–N str, Amide III			
1334			Glycogen	
1338	Trp			G
1366		Sym. str. CH_3_		
1381			Glycogen	
1450	CH_2_ def	CH_2_ def		
1485	CH_2_ def			G, A
1560	Tyr, Trp			
1580				A, G ring br
1584	C=C str, C=C bend. Trp, Phe			
1605	C=C Phe, Tyr			
1642	C=O str, C=C sym. str.			
1669	C=O str. Amide I			

**Table 3:** Classification of blinded test set samples (*n* = 14) as transcriptionally active or non-transcriptionally active by (1) testing each cellular spectrum individually and (2) testing the mean spectrum of each sample.

Sample	Correct Classification	Individual Cellular Spectra		Mean Sample Spectra	
		Non-Transcriptionally Active	Transcriptionally Active	Non-Transcriptionally Active	Transcriptionally Active
001	Transcriptionally active	0	16	0	1
002	Transcriptionally active	15	16	0	1
003	Transcriptionally active	1	12	0	1
004	Transcriptionally active	4	33	0	1
005	Transcriptionally active	7	25	0	1
006	Transcriptionally active	0	19	0	1
007	Transcriptionally active	2	17	0	1
008	Transcriptionally active	3	16	0	1
009	Transcriptionally active	4	44	0	1
010	Transcriptionally active	10	6	1	0
011	Non-transcriptionally active	29	0	1	0
012	Non-transcriptionally active	26	3	1	0
013	Non-transcriptionally active	21	3	1	0
014	Non-transcriptionally active	22	2	1	0

**Table 4:** Raman classification, cytology results, histology results, and HPV testing results for the blinded test set (*n* = 14).

Sample Number	Raman Classification	Cytology	Histology	HPV DNA	HPV mRNA
001	Transcriptionally active	HSIL	CIN2	Positive	Positive
002	Transcriptionally active	HSIL	CIN3	Positive	Positive
003	Transcriptionally active	HSIL	CIN3	Positive	Positive
004	Transcriptionally active	HSIL	CIN2	Positive	Positive
005	Transcriptionally active	HSIL	CIN1	Positive	Positive
006	Transcriptionally active	HSIL	CIN2	Positive	Positive
007	Transcriptionally active	HSIL	CIN2	Positive	Positive
008	Transcriptionally active	HSIL	CIN3	Positive	Positive
009	Transcriptionally active	HSIL	CIN2	Positive	Positive
010	Non-transcriptionally active	LSIL	CIN1	Positive	Positive
011	Non-transcriptionally active	LSIL	Negative	Positive	Negative
012	Non-transcriptionally active	Negative	Negative	Positive	Negative
013	Non-transcriptionally active	LSIL	Negative	Positive	Negative
014	Non-transcriptionally active	Negative	Negative	Positive	Negative

## Data Availability

The data that support the findings of this study are available from the corresponding author upon reasonable request.
